# Metabolite abundance in bovine preovulatory follicular fluid is influenced by follicle developmental progression post estrous onset in cattle

**DOI:** 10.3389/fcell.2023.1156060

**Published:** 2023-05-05

**Authors:** Emma A. Hessock, J. Lannett Edwards, F. Neal Schrick, Rebecca R. Payton, Shawn R. Campagna, Abigayle B. Pollock, Hannah M. Clark, Allyson E. Stokes, Jessica L. Klabnik, Kennedy S. Hill, Samantha R. Roberts, Meredith G. Hinson, Sarah E. Moorey

**Affiliations:** ^1^ Department of Animal Science, University of Tennessee, Knoxville, TN, United States; ^2^ Department of Chemistry, University of Tennessee, Knoxville, TN, United States

**Keywords:** preovulatory follicle, follicular fluid, metabolome, cow, estrus, ovulation

## Abstract

**Introduction:** Preovulatory follicle response to the luteinizing hormone (LH) surge leads to metabolic, molecular, and functional changes in the oocyte and somatic follicular cells from the onset of estrus to ovulation. Follicular fluid contains metabolites, miRNAs, proteins, and hormones that are byproducts of follicular metabolism and support cellular processes of oocyte, cumulus, and granulosa constituents. Numerous studies have highlighted the importance of follicular fluid composition to support fertility, but critical gaps exist toward understanding dynamic modifications in the follicular fluid metabolome from estrous onset to ovulation. The hypothesis was that abundance of follicular fluid metabolites is dependent on follicle progression post LH surge and variability in follicular fluid metabolome profiles indicate key processes required for preparation of the follicle and oocyte for optimal fertility. The objective was to generate preovulatory follicular fluid metabolome profiles and discern differences in the metabolome of preovulatory follicular fluid samples collected at onset of estrus, 11 h post estrous onset, and 18 h post estrous onset.

**Methods:** Estrus was synchronized in non-lactating Jersey cows (n=40) and follicular fluid was collected immediately after the first observed standing mount (hr 0) or at approximately h 11 or 18 after the first standing mount. Ultra-High-Performance Liquid Chromatography-High Resolution Mass Spectrometry was performed on preovulatory follicular fluid samples (*n* = 9 collected at hr 0, 9 at h 11, and 10 at h 18) and a multiple linear model was performed to determine if time post estrous onset impacted metabolite abundance.

**Results:** Metabolites influenced by time post estrous onset were tested for enrichment in KEGG pathways. Ninety metabolites were identified in follicular fluid samples. Twenty metabolites differed in abundance among timepoints post estrous onset (*p* ≤ 0.05). Pathways corresponding to amino acid and energy metabolism were enriched with metabolites impacted by time post estrous onset (FDR ≤ 0.10).

**Discussion:** Results from the current study indicate early response to the LH surge to increase bioavailability of amino acids and metabolites used by the cumulus and granulosa cells for energy production and shuttled into the oocyte to support meiotic maturation. Such metabolites may later be used by the ovulatory follicle for protein production.

## 1 Introduction

Optimal fertility in cattle, humans, and other mammalian species relies on dynamic transformations in the oocyte, preovulatory follicle’s somatic cells, and follicular fluid milieu during the days leading up to estrus and the hours between estrus and ovulation. In bovine, estrous onset is a key developmental milestone in the follicle’s progression toward ovulation. This is because the preovulatory gonadotropin surge closely follows onset of estrus (Dieleman et al., 1986; Rajamahendran et al., 1989; Stevenson et al., 1998; Mondal et al., 2006) and signals the cascade of events within the follicle that commence in the ovulation of a mature oocyte from a follicle, that is, transitioning into a functional corpus luteum (Richards et al., 1998; Duffy et al., 2018; Robker et al., 2018). Specifically, the luteinizing hormone (LH) surge serves as the stimulant for the onset of oocyte maturation, follicle cell luteinization, increased follicular vascularity, and breakdown of the follicle wall that leads to ovulation (Richards et al., 1998; Duffy et al., 2018; Robker et al., 2018). In cattle that displayed estrus, the first elevation of circulating LH concentrations that was followed by higher LH values was observed to occur approximately 2 h after the onset of estrus, and the LH surge peak was reported at approximately 4.5 h after the onset of estrus (Dieleman et al., 1983).

Progression of the follicle and oocyte toward ovulation can be roughly divided into intrafollicular events occurring pre-LH surge exposure, in early response to the LH surge, and in later response to the LH surge as ovulation nears. Before the LH surge, follicular granulosa cells remain in a proliferative phase. The oocyte and surrounding cumulus cells are intimately connected to each other via paracrine signaling and direct transfer of metabolites, RNAs, and signaling molecules such as cyclic AMP (cAMP) and cyclic GMP through gap junctional complexes that span the zona pellucida (Eppig, 1985; Gilchrist et al., 2004; Macaulay et al., 2014). Rising LH levels associated with the preovulatory gonadotropin surge act through binding of receptors on the granulosa cells to initiate ovulatory events. Granulosa cells cease dividing and undergo differentiation in preparation for future secretory roles in the corpus luteum, their metabolism and the synthesis of amino acids increases, and granulosa mediated pathways signal for follicular angiogenesis and inflammation. Cumulus cell expansion, breakdown of gap junctional transfer from the cumulus cells to the oocyte, and a drop in cAMP in the oocyte that leads to onset of oocyte nuclear maturation also result from granulosa cells mediated response to the LH surge (Eppig, 1985; Granot and Dekel, 2002; Norris et al., 2009). The dramatic follicular response to the LH surge ranges from changes in gene expression that take place almost immediately to biological processes that occur over multiple hours. Physically identifiable differences in oocyte maturation have been reported at approximately 9–12 and 18–20 h after the peak LH surge. Disconnection of junctions between the oocyte and cumulus cells, alongside breakdown of the oocyte nucleus, was first reported between 9 and 12 h after the peak LH surge (Callesen et al., 1986; Hyttel et al., 1986). By 15 h after peak LH surge, spatial rearrangements of organelles were observed in the oocyte’s cytoplasm (Hyttel et al., 1986), and by 18–20 h after peak LH surge the first polar body was extruded (Callesen et al., 1986; Hyttel et al., 1986). Similarly, granulosa cells exhibit a time dependent response to the LH surge. Leading up to estrus, and the LH surge, follicular estradiol production rises to peak around the onset of estrus (Henricks et al., 1971; Wettemann et al., 1972; Ginther et al., 2013). However, 6 h post estrous onset, intrafollicular hormone concentrations of estradiol and progesterone have abruptly shifted to favor progesterone production. Accumulation of members of the vascular endothelial cell family of growth factors to promote angiogenesis (Duffy et al., 2018) and production of the enzyme prostaglandin synthase two that catalyzes conversion of membrane lipids to prostaglandin H2 (Richards, 1997) also occur quickly after the LH surge.

The currently known post-LH surge intrafollicular changes in steroid hormone profiles, somatic molecular blueprint, cumulus-oocyte association, and oocyte maturation likely lead to or are accompanied by further transient modifications in the follicular fluid to further promote optimal fertility. In recent years, much progress has been made toward understanding the follicular fluid’s composition and role in fertility. This highly complex biofluid contains numerous molecules and components such as enzymes, amino acids, extracellular vesicles, hormones, proteins, and lipids (Revelli et al., 2009; Ambekar et al., 2013). A number of studies have linked abundance of specific proteins, metabolites, and mRNAs to roles in the follicle that support oocyte developmental competence and pregnancy outcome (Leroy et al., 2005; Bender et al., 2010; Hung et al., 2015; Kafi et al., 2017; Bou Nemer et al., 2019; Rispoli et al., 2019; Afedi et al., 2021; Read et al., 2021; Read et al., 2022). Related to cycle specific impacts on the follicular fluid metabolome, composition naturally varies across the estrous cycle in cows (Orsi et al., 2005), but no known studies have examined post LH surge progression on the bovine follicular fluid metabolome.

Given the importance of the follicular fluid metabolic constituents and known transitions in follicular cell function and metabolism during the time from estrus to ovulation, it is essential to further characterize time specific modifications in the follicular fluid metabolome of bovine preovulatory follicles from the time of estrous onset throughout oocyte and follicle maturation. The hypothesis of this study was that abundance of follicular fluid metabolites is dependent on follicle progression post LH surge and that variability in follicular fluid metabolome profiles indicate key processes required for optimal preparation of the follicle and oocyte for optimal fertility post ovulation. Therefore, a study was performed with the objective to generate preovulatory follicular fluid metabolome profiles and discern differences in the metabolome of preovulatory follicular fluid samples collected at onset of estrus, 11 h post estrous onset, and 18 h post estrous onset.

## 2 Materials and methods

### 2.1 Experimental design

The primary objective of this study was to determine time specific modifications in the metabolome of preovulatory follicular fluid collected from cattle at the onset of estrus to approximately 18 h after estrous onset. Estrus was synchronized in 40 non-lactating Jersey cows (mean age = 5.21 years; mean lactations = 1.61) and the preovulatory follicle’s fluid was collected either immediately after the first observed standing mount (hr 0) or at approximately hr 11 or 18 after the first observed standing mount. Ultra-High-Performance Liquid Chromatography - High Resolution Mass Spectrometry (UHPLC-HRMS) was performed on preovulatory follicular fluid samples that were successfully collected at either hr 0, hr 11, or hr 18 after an animal first displayed standing estrus. Metabolites were identified and quantified using Metabolomic Analysis and Visualization Engine (MAVEN). Prior to statistical analyses, samples were filtered based on follicular fluid estradiol to progesterone ratio to ensure that follicles were non-atretic and of hormone profiles expected for their assigned stage post estrous onset. Statistical analyses were performed on 8 samples collected at hr 0, 6 samples collected at hr 11, and 6 samples collected at hr 18. Sparse Partial Least Squares Discriminant Analysis (sPLS-DA) was performed to allow visual clustering of samples based on time post estrous onset. A step down multiple linear model, followed by analysis of variance (ANOVA; type III sum of squares) was performed on all metabolites to determine if time post estrous onset impacted the abundance each follicular fluid metabolite. Tukey’s Honest Significant Difference *post hoc* test (Tukey’s HSD) was conducted to determine pairwise differences between hr 0 vs. hr 11, hr 0 vs. hr 18, and hr 11 vs. hr 18 follicular fluid metabolites. Metabolites influenced by time post estrous onset were then tested for enrichment in Kyoto Encyclopedia of Genes and Genomes (KEGG) pathways.

### 2.2 Synchronization of estrus and follicular development

**FIGURE 1:**
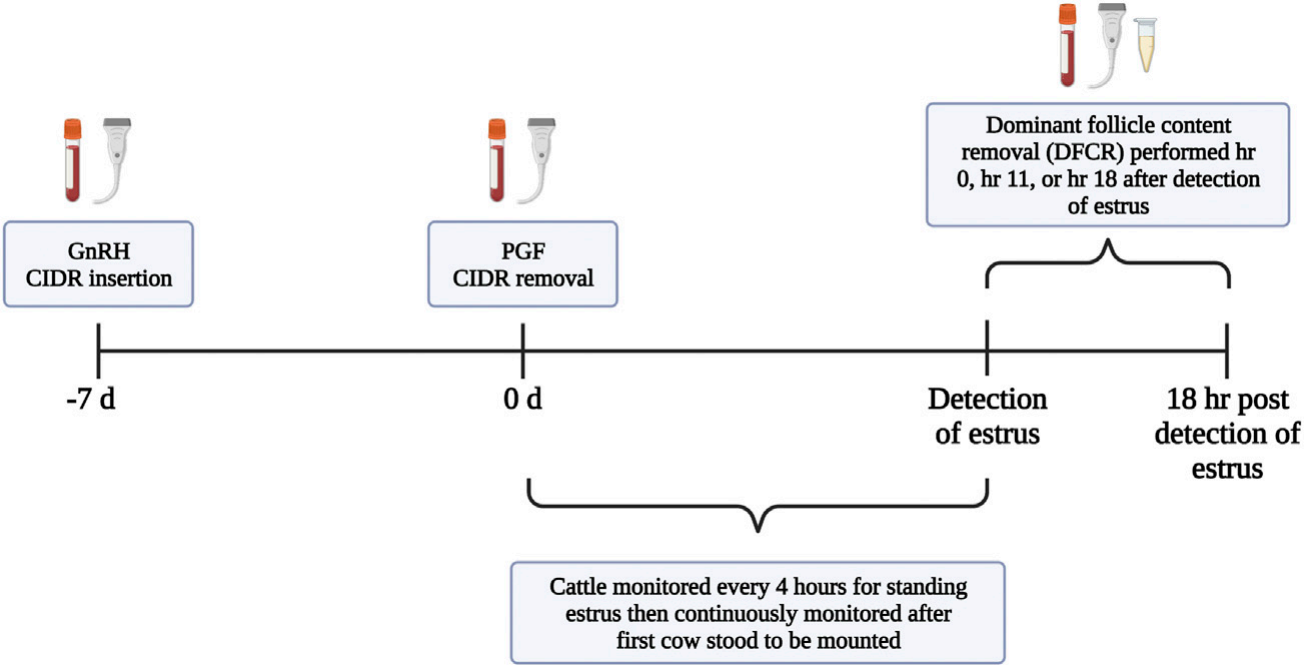
Timeline for synchronization of estrus and follicular fluid collection.

All protocols and procedures were approved by the Institutional Animal Care and Use Committee at the University of Tennessee. Non-lactating Jersey cows (*n* = 40; mean age = 5.21 years; mean lactations = 1.61) which were maintained as a grazing herd at one of the University of Tennessee AgResearch and Education Centers underwent pre-synchronization, synchronization of estrus, and dominant follicle content removal (DFCR; Figure 1). Throughout the study’s duration cattle were provided with *ad libitum* access to fescue-based pasture, hay, and water. Cows were maintained in multiple, interconnected large pens where the gates remained open to allow for free movement of the animals. Cows were moved to an adjacent working area to perform all procedures and upon completion were returned back to the pens where they remained until completion of the study. Pre-synchronization was performed by administration of gonadotropin releasing hormone (GnRH; Cystorelin^®^; 100 ug; i.m.; Boehringer Ingelheim; Ingelhein am Rhein, Germany) and insertion of a controlled internal drug release (CIDR intervaginal insert; 1.38 g progesterone; Eazi-Breed™ CIDR^®^; Zoetis Animal Health, Kalamazoo, MI, United States). Nine days later, the CIDR was removed and Prostaglandin F2⍺ (PGF; 12.5 mg of dinoprost tromethamine/mL Lutalyse^®^ HighCon; Zoetis Animal Health, Kalamazoo, MI, United States) was administered. Forty-8 hr after PGF administration and CIDR removal, when animals were expected to be in the follicular phase of the estrous cycle, cows were administered GnRH and a CIDR was inserted (d-7). Seven days later (d0) the CIDR was removed, PGF was administered, and an Estrotect™ patch (Estrotect; Rockway Inc.; spring Valley, WI, United States) was adhered to the tailhead. Cows were visually monitored for estrus every 4 h in a pen containing pasture and dry dirt areas until estrous activity began, then monitoring occurred continuously. All personnel were trained in visual observation of estrus based on standing heat. Patches were used to assist with visual detection of estrus but were not the sole or main method to detect estrus. Patches were scored using a 1–4 scale (score 1 = <25% rubbed off, score 2 = 25%–50% rubbed off, score 3 = 50%–75% rubbed off, score 4 = >75% rubbed off). Secondary signs of estrus (mounting, riding other cows, and chin resting on the tailhead) were also recorded, and onset of estrus was defined as the time of first recorded instance of an animal visually standing to be mounted. Cattle underwent DFCR via transvaginal ultrasound guided aspiration either within 2 h 55 min of first observed standing mount (hr 0; 1 h 47 ±15 min; 11 min–2h 55 min) or between 10 h 18 min–12 h 50 min (hr 11; 11 h 29 min ±16 min) or 17 h 49 min–18 h 44 min (hr 18; 18 h 14 min ±5 min) after first observed standing mount.

### 2.3 Blood collection, ovarian mapping, cow weight, and body condition

Blood was collected and ovarian status recorded at the time of GnRH administration and CIDR insertion on d −7, CIDR removal and PGF administration on d 0, onset of estrus, and when DFCR occurred. Blood was collected via venipuncture in the coccygeal vein of the tail, and approximately 10 mL of blood was collected into BD Vacutainer^®^ serum collection tubes using BD Vacutainer^®^ single-sample 18-gauge 1-inch needles attached to a collar (BD, Franklin Lakes, NJ, United States). Blood samples remained at room temperature for 1 h to allow for clotting to occur. Samples were then stored at 4°C for 24 h before undergoing centrifugation at 1,200 x g for 25 min at 4°C. Serum was collected and stored in borosilicate glass tubes at −20°C for hormone analysis. Transrectal ultrasonography was performed using an IBEX EVO^®^ II ultrasound and the eL7 linear probe (E.I Medical Imaging, Loveland, CO, United States). For both ovaries, follicles >7 mm were measured in millimeters using the average of the largest diameter of the follicle and the diameter perpendicular to the largest diameter. Other notable structures such as small cohorts of follicles and all corpora lutea were recorded. Animal weight (kg) and body condition score were assessed at the onset of synchronization, and records for age at DFCR were acquired. Body condition score was measured using the dairy body condition score scale of 1–5 where a score of 1 indicates severe under condition and 5 indicates severe over condition; (Wildman et al., 1982).

### 2.4 Dominant follicle content removal via transvaginal aspiration

After estrus was detected, cattle underwent DFCR at hr 0, hr 11, or hr 18 via transvaginal aspiration which was performed by a trained technician (Read et al., 2021; Horn et al., 2022; Read et al., 2022). Cows were administered a spinal block using 5 mL of 2% lidocaine in the first intercoccygeal space of the tailhead prior to transvaginal aspiration. The perineal and vulvar area was thoroughly cleaned to remove potential contaminants from entering the vagina. An ultrasound guided transvaginal aspiration apparatus encompassing a CFA-9 convex ultrasound probe connected to a Samsung HM70A ultrasound, 18-gauge spinal needle, and plastic tubing for follicular fluid collection was inserted into the anterior vagina. Using the ultrasound for visualization, the ovary was manipulated until the follicle of interest was in the best position for aspiration. The needle was advanced through the vaginal wall and ovarian cortex until the tip was inserted in the follicular antrum. Follicular fluid was aspirated into a 5 mL syringe until the follicle was visualized to have fully collapsed. The transvaginal aspiration device was cleaned with chlorhexidine and rinsed with water, and all tubing was completely flushed with sterile water between each cow.

### 2.5 Processing of follicular fluid

After aspiration, follicular fluid in the 5 mL syringe was distributed into one well of a 4 well Petri dish. A trained technician searched the dish under a microscope for the cumulus oocyte complex, which if found was removed and collected for future use in a different study. The follicular fluid was collected from the 4 well Petri dish and distributed into 1.7 mL Eppendorf tubes which were centrifuged at 500 x g for 5 min at 4°C to pellet potential cellular debris within the sample. Supernatant was aliquoted into multiple tubes, snap frozen in liquid nitrogen, and stored at −80°C for future analyses. Specifically, 60 μL of follicular fluid supernatant was placed in a 2 mL microcentrifuge tube for metabolomic analyses, 100 μL was placed in a 2 mL microcentrifuge tube for follicular fluid estradiol and progesterone assays, and remaining follicular fluid was divided amongst 2 mL polypropylene cryovials (Stellar Scientific, Baltimore, MD, United States) for procedures outside the scope of this study.

### 2.6 Profiling of the follicular fluid metabolome

Ultra-High-Performance Liquid Chromatography—High Resolution Mass Spectrometry (UHPLC-HRMS) was performed on follicular fluid samples at the Biological and Small Molecule Mass Spectrometry Core (RRID: SCR_021368) at the University of Tennessee, Knoxville. Sixty μL aliquots of follicular fluid samples (*n* = 29) were thawed at room temperature, and metabolites within each sample were extracted using a 40:40:20 methanol/acetonitrile/water solution with 0.1 M formic acid following a previously reported procedure (Rabinowitz and Kimball, 2007; Lu et al., 2010). An extraction solvent was added to the samples which underwent agitation and vortexing before samples were then chilled at −20°C for 20 min. Once samples were properly chilled, the tubes were centrifuged to form pellets and remove debris from the sample. A second set of 2 mL microcentrifuge tubes were used to collect the supernatant from the first set of tubes. The extraction solvent was again dispensed into the first set of tubes, which contained the pellet, and the mixture was pipetted to resuspend the pellet. The tubes were then re-submitted to agitation and vortexing, chilling, and centrifugation as described above. The supernatant from the tube was added to the second set of tubes which contained the previous supernatant. The first tube set with the final pellet was then discarded. The second set of tubes containing the supernatant underwent drying using nitrogen gas. Once dried, tubes were filled with MilliQ water to resuspend samples and moved to new autosampler vials. Metabolomic samples were separated using columns (Synergi Hydro RP, 2.5 μm, 100 mm × 2.0 mm column; Phenomenex, Torrance, CA, United States) which were maintained at 25°C. Solvents to elute metabolites during the mobile phase were 1) 97:3 methanol/water with 15 mM acetic acid and 11 mM tributylamine and 2) 100% methanol. At a flow rate of 0.2 μL/min, the solvent gradient was 1) 100% and 2) 0% from 0 to 5 min, 1) 80% and 2) 20% from 5 to 13 min, 1) 45% and 2) 55% from 13 to 15.5 min, 1) 5% and 2) 95% from 15.5 to 19 min, and 1) 100% and 2) 0% from 19 to 25 min. An Exactive Plus Orbitrap mass spectrometer (Thermo Fisher Scientific, Waltham, MA, United States) with an electrospray ionization probe attached was used, operating in negative mode with a scan range between 72 and 1,000 m/z, a resolution of 140,000, and an acquisition gain control of 3 × 10^6^.

### 2.7 Initial analysis of metabolome data

The mass spectrometry generated Xcalibur (RAW) files were converted to an open source mzML format (msconvert software; ProteoWizard package). The converted files were processed using the Metabolomic Analysis and Visualization Engine (MAVEN; mzroll software, Princeton University) to complete an untargeted analysis of the liquid chromatography mass spectrometry data. MAVEN identifies metabolites using a variety of factors including peak shape, retention time, and signal-to-noise-ratio. The program then generated pre-processed peak data tables which were used for statistical analysis.

### 2.8 Follicular fluid and serum hormone profile evaluation

Serum and follicular fluid progesterone concentrations were evaluated using the ImmuChem progesterone double antibody radioimmunoassay kit (MP Biomedicals, Costa Mesa, CA, United States) and following manufacturer’s specifications. Serum progesterone intra- and inter-assay coefficients of variation (CV) were 8.00% and 4.18%, respectively. Follicular fluid progesterone intra- and inter-assay CVs were 4.42% 5.70%, respectively. Serum estradiol concentration was determined using methods previously validated and described (Kirby et al., 1997). Serum estradiol intra- and inter-assay CVs were 3.55% and 6.70%, respectively. Follicular fluid samples underwent dilution from 1:2500 to 1:50000 before follicular fluid estradiol concentration was quantified using The DetectX^®^ Serum 17β-Estradiol ELISA Kit (Arbor Assays, Ann Arbor, MI, United States) according to manufacturer’s specifications. Intra- and inter-assay CVs for follicular fluid estradiol were 1.65% and 7.33%, respectively.

### 2.9 Statistical analysis

All statistical procedures were performed using R Studio version 4.1.2, (R Team, 2020), or MetaboAnalyst 5.0 (Pang et al., 2021). Data was first processed to remove samples that lacked hormone data on follicular fluid due to inadequate volume (*n* = 1 at h 11 and 2 at h 18) or had follicular fluid estradiol to progesterone (FF E2:P4) ratios indicative of potentially less or more advanced progression post estrous onset (Fortune and Hansel, 1985) compared to their cohort and assigned time (*n* = 2 at h 0, 2 at h 11, and 2 at h 18). Though this procedure reduced sample numbers to 7 samples at hr 0, 6 samples at hr 11, and 6 samples at hr 18, it was essential to enhance confidence in time classification by combining visual estrous observation efforts with confirmation of follicular fluid hormones. Such procedures removed samples with FF E2:P4 ratios that overlapped with other timepoints and that may have originated from animals first detected in estrous hours before or after first actual mount and who would have been at variable progression relative to estrous onset compared to other animals within their treatment cohort. Samples removed from hr 0 based on FF E2:P4 ratio had ratios of 7.22 and 9.03, compared to hr 0 cohorts with ratios of 17.1–65.07. Samples removed from hr 11 based on ratio had FF E2:P4 ratios of 3.12 and 14.27, compared to hr 11 cohorts with ratios from 4.47 to 9.35. Samples removed from hr 18 based on FF E2:P4 ratio had ratios of 0.2 and 15.9, compared to hr 18 cohorts that ranged from 2.51 to 3.77.

Mean age, body condition score, serum estradiol concentration at follicle collection, and serum progesterone concentration at follicle collection were calculated, and differences among timepoints post estrous onset were determined using ANOVA. The effect of timepoint was considered significant if *p* ≤ 0.05. Post hoc comparisons between hr 0 vs. hr 11, hr 0 vs. hr 18, and hr 11 vs. hr 18-time groups were performed using the “emeans” function in R, which utilized Tukey’s HSD and provided least squares means for trait analyzed at each time point. Differences between phenotypic timepoint comparisons were considered significant if Tukey’s HSD *p* ≤ 0.05.

Peak area data for samples selected for statistical analyses was then input into MetaboAnalyst 5.0, filtered for relative standard deviation, and log transformed before performing sPLS-DA and plotting samples based on 2 components and 10 variables per component (Lê Cao et al., 2011). Ninety-five percent confidence intervals were then calculated and drawn around clustered samples from each timepoint.

Using R Studio, data were tested for normality using Shapiro Wilke test, and metabolites that failed normality assumption were log transformed. Due to detection of outliers for metabolite abundance within treatment (hr 0, hr 11, hr 18) that hindered achieving normality and influenced statistical results, samples with outlier metabolite abundance compared to their time group were removed for analyses of the specific metabolite in which the outlier was identified. Initial linear models were performed to detect potential relationships between abundance of each metabolite and independent variables of cow age, weight, body condition score (BCS), serum and follicular fluid steroid hormone concentrations, and follicle diameter. We then designed a multiple linear model for each metabolite with metabolite abundance as the dependent variable and time group (hr 1, hr 11, hr 18) alongside the above variables with *p* < 0.10 as independent variables. Covariates were removed from the model in a stepwise fashion with the least significant variable removed each time until only covariates where *p* ≤ 0.05 remained in the final model alongside the variable of interest (time group). If time group was significant (*p* ≤ 0.05) based on the ANOVA function utilizing Type III Sum of Squares, *post hoc* comparisons between hr 0 vs. hr 11, hr 0 vs. hr 18, and hr 11 vs. hr 18-time groups were performed using the “emeans” function in R, which utilized Tukey’s HSD and provided least squares means for metabolite abundance at each time point. Differences between timepoint comparisons were considered significant or trending if Tukey’s HSD *p* ≤ 0.05 or 0.05 ≤ *p* ≤ 0.10, respectively.

Three KEGG pathway enrichment analyses of metabolites influenced by time post estrous were performed using MetaboAnalyst 5.0 with the *Bos taurus* reference metabolome. Based on results of metabolite statistical analysis, 3 lists of metabolites influenced by time post estrous onset were interrogated for pathway enrichment analysis: 1) all metabolites that differed among timepoints post estrous onset, 2) metabolites that rose in abundance from hr 0 to hr 11, and 3) metabolites that rose in abundance from hr 0 to hr 11 and then decreased in abundance from hr 11 to hr 18. Pathways were reported if false discovery rate (FDR) ≤ 0.10.

A secondary analysis was performed that classified samples into categories based on FF E2:P4 ratio. Samples with FF E2:P4 ratio >10 were classified into a cohort based on FF E2:P4 expected at onset of estrus. Samples with FF E2:P4 ratio >4 and <10 were classified into a second cohort based on FF E2:P4 expected approximately 11 h post onset of estrus, and samples with FF E2:P4 ratio <4 were categorized into a third cohort based on FF E2:P4 expected approximately 18 h post onset of estrus. Statistical analyses were performed as described above to determine relationships between FF E2:P4 category and metabolome profiles. Metabolites that differed among FF E2:P4 classifications were utilized to perform 3 KEGG pathway enrichment analyses, as described above, utilizing lists of all differentially abundance metabolites, metabolites that rose from FF E2:P4 classification indicative of estrous onset to FF E2:P4 classification indicative of hr 11 post estrous onset, and metabolites that rose from FF E2:P4 classification indicative of estrous onset to FF E2:P4 classification indicative of hr 11 post estrous onset and then decreased in abundance from FF E2:P4 classification indicative of hr 11 post estrous onset to FF E2:P4 classification indicative of hr 18 post estrous onset.

## 3 Results

### 3.1 Description of cow phenotypes, hormone profiles, and preovulatory follicular fluid metabolome profiles at 0, 11, and 18 h post onset of estrus

Mean age and BCS of cows classified and included in analyses as hr 0, 11, or 18 post estrous onset was 2012 ± 19 days and 3.11 ± 0.07, respectively. Mean serum progesterone concentration at the time of dominant follicle content removal was 0.10 ± 0.02 ng/mL. Neither age (*p* = 0.96), BCS (*p* = 0.49), or serum progesterone at follicle content removal (*p* = 0.28) differed among collection timepoints. Mean serum estradiol concentration at the timepoint of dominant follicle content removal was 6.62 ± 0.02 pg/mL. Serum estradiol concentration differed among collection timepoints (*p* = 0.037), such that samples collected at hr 18 had lower serum estradiol concentration than samples collected at hr 0.

**FIGURE 2:**
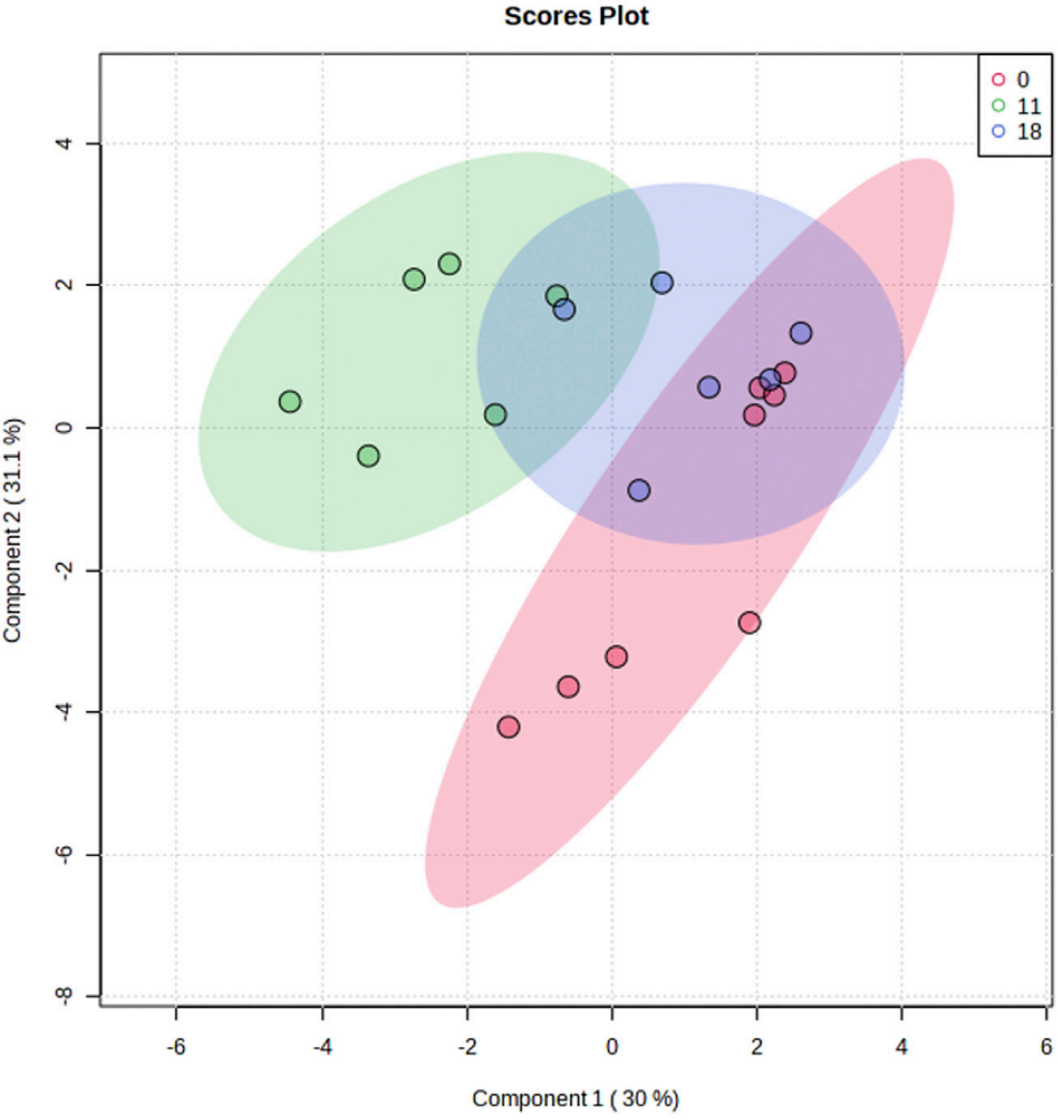
Sparse Partial Least Squares Discriminant Analysis of follicular fluid metabolome profiles from preovulatory follicular fluid samples collected at onset of estrus (hr 0), hr 11 after estrous onset, or hr 18 after estrous onset. Shaded ellipses indicate 95% confidence area around each timepoint cluster.

Ninety metabolites were identified in preovulatory follicular fluid samples collected at onset of estrous and hr 11 and 18 following estrous onset. Metabolites consisted primarily of amino acids, byproducts of glucose metabolism and the tricarboxylic acid (TCA) cycle, and varied other small molecules such as myo-Inositol, Cholate, and the nucleosides Uridine and Deoxyuridine. Plotting of samples utilizing sPLS-DA revealed independent clustering of samples collected at the onset of estrous from those collected 11 h post estrous onset (Figure 2). Samples collected at 18 h post estrous onset clustered such that their 95% confidence interval intersected confidence intervals surrounding both hr 0 and 11 samples. While most hr 11 and 18 samples clustered tightly, hr 0 samples dispersed along the *y*-axis (Component 2) to form two subclusters. Further interrogation of hr 0 samples belonging to each subcluster did not reveal visually identifiable differences in cow phenotype, follicle dynamics, or hormone profiles between the two subgroups.

### 3.2 Preovulatory follicular fluid metabolites influenced by time post estrous onset

**FIGURE 3:**
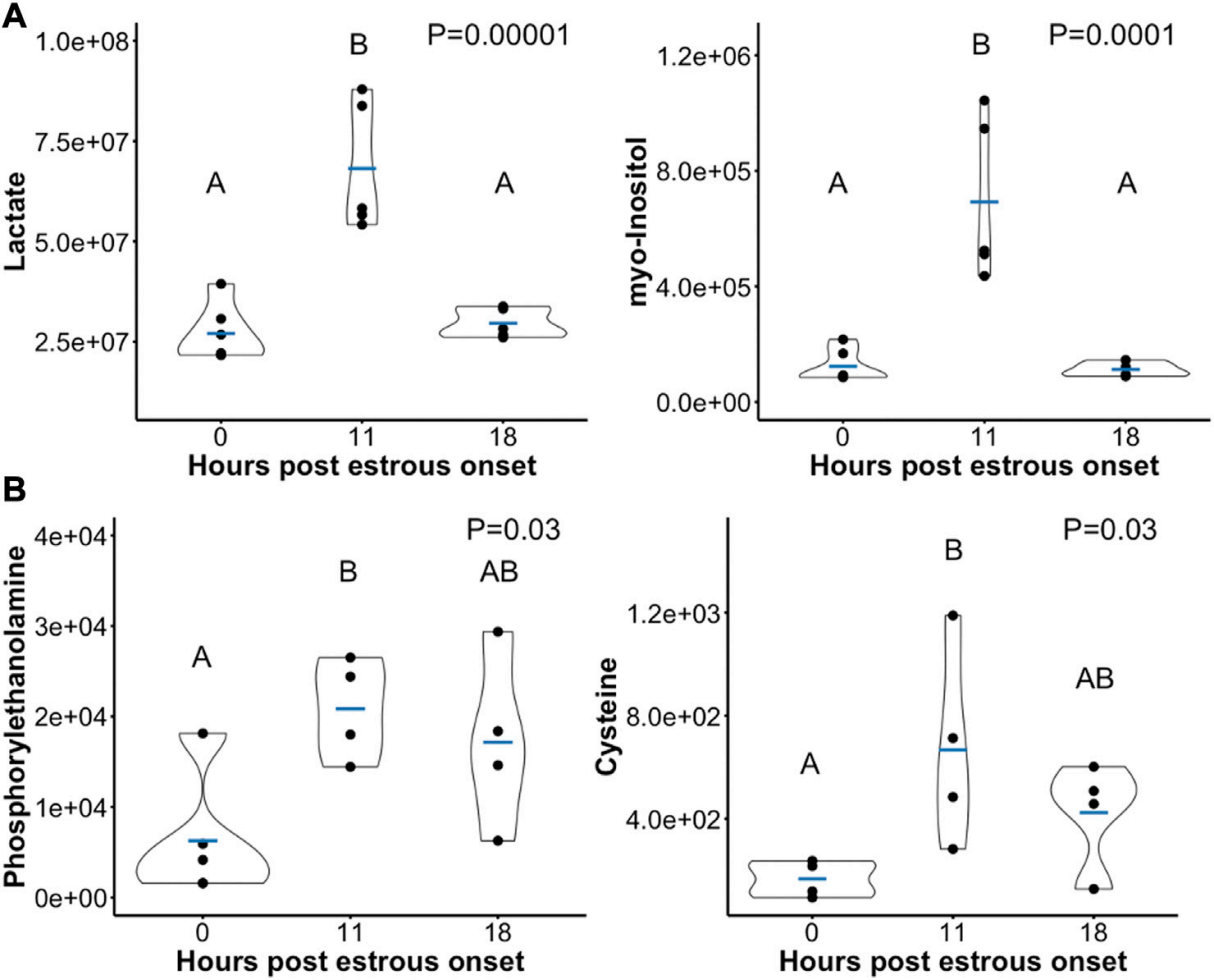
Scatterplots depicting peak area (*y*-axis) of example preovulatory follicular fluid metabolites that rose in abundance from hr 0 to hr 11 post onset of estrus. A subset of metabolites dropped in abundance after hr 11 such that metabolite levels at hr 18 were significantly lower than at hr 11 **(A)**. In other metabolites, abundance was not different between hr 18 and either hr 0 or hr 11 **(B)**. Mean metabolite abundance at timepoints with different letters are statistically different (Tukey’s Honest Significant Difference *post hoc* test *p* ≤ 0.05).

Of the 90 metabolites identified in preovulatory follicular fluid, 20 differed in abundance based on time post onset of estrus (Table 1). The most profound finding was that 17 of these 20 metabolites rose in abundance from hr 0 to hr 11 after first instance of standing estrus (myo-Inositol, Lactate, Pyruvate, N-acetyl-D-glucosamine 6-phosphate, Alpha-Ketoglutarate, Cholate, Glycerol-3-phosphate, Hydroxyphenylacetate, Deoxyuridine, Orotate, Xylitol, Aconitate, 3-Hydroxyisovalerate, Kynurenine, Cysteine, Phosphorylethanolamine, Glutamate; Figure 3).

**TABLE 1:** Preovulatory follicular fluid metabolites that varied in abundance among collection times post estrous onset.

**Metabolite**	**^†^** **Least squares means**			
	**0**	**11**	**18**	* **P**
myo-Inositol	123,311 ± 27,711^a^	692,126 ± 33,253^b^	112,739 ± 41,567^a^	<0.001
Lactate	27,092,488 ± 1,705,098^a^	68,150,779 ± 2,046,118^b^	29,618,133 ± 2,046,118^a^	<0.001
Pyruvate	4,494,127 ± 210,682^a^	7,303,056 ± 245,795^b^	4,123,317 ± 245,795^a^	0.003
N-acetyl-D-glucosamine 6-phosphate	1.7 ± 264^a^	2,183 ± 264^b^	82 ± 329^a^	0.003
Alpha-Ketoglutarate	39,869 ± 4,353^a^	98,500 ± 5,079^b^	31,092 ± 5,079^a^	0.003
Cholate	24,199 ± 5,584^a^	63,485 ± 3,722^b^	19,617 ± 4,467^a^	0.0030
Lysine	43,270 ± 2,618^a^	11,162 ± 3,055^b^	22,285 ± 3,055^ab^	0.005
Glycerol-3-phosphate	1,099 ± 1,399^a^	13,002 ± 1,166^b^	4,860 ± 1,166^Ab^	0.006
Hydroxyphenylacetate	91,242 ± 8,226^a^	167,493 ± 5,965^b^	117,551 ± 5,431^ab^	0.013
Deoxyuridine	324 ± 167^a^	1,536 ± 151^b^	549 ± 138^Ab^	0.013
Aspartate	76,716 ± 4,158^a^	107,629 ± 4,511^ab^	123,471 ± 4,103^b^	0.020
Xylitol	67,279 ± 1,863^a^	87,249 ± 2,235^b^	69,446 ± 2,235^aB^	0.020
2,3-Dihydroxybenzoate	10,828 ± 914^ab^	16,255 ± 914^b^	8,090 ± 914^a^	0.020
Aconitate	32,802 ± 1,659^a^	46,684 ± 1,659^b^	33,734 ± 1,105^a^	0.023
3-Hydroxyisovalerate	1,548,328 ± 30,540^a^	1,788,369 ± 20,360^b^	1,612,064 ± 30,540^ab^	0.025
Kynurenine	8,059 ± 1,324^a^	19,025 ± 1,324^b^	6,593 ± 1,589^aB^	0.031
Cysteine	166 ± 64^a^	667 ± 54^b^	424 ± 64^ab^	0.034
Orotate	1,402 ± 626^a^	5,719 ± 522^b^	818 ± 522^a^	0.015
Phosphorylethanolamine	6,279 ± 1,495^a^	20,837 ± 1,869^b^	17,154 ± 1,869^ab^	0.036
Glutamate	116,870 ± 16,746^a^	28,0252 ± 16,746^b^	197,926 ± 16,746^ab^	0.041

^†^ Least squares mean ± standard error of the mean for metabolite peak area.

^*^ P value reported for main effect of time classification.

^ab^ P ≤ 0.05.

^aA or bB^ P ≤ 0.10. Pairwise comparison *p* values were adjusted using Tukey's Honest Significant Difference post hoc test.

Aside from Deoxyuridine and Glycerol-3-phosphate which tended to remain elevated at hr 18 compared to hr 0, all other metabolites that initially rose were no longer different in abundance between hr 0 and 18 post estrous onset. Ten metabolites declined or tended to decline in abundance from hr 11 to 18 (myo-Inositol, Lactate, Pyruvate, N-acetyl-D-glucosamine 6-phosphate, Alpha-Ketoglutarate, Cholate, Orotate, Xylitol, Aconitate, Kynurenine; Figure 3A). The 5 remaining metabolites that rose from hr 0 to hr 11 remained at a level such that no differences were observed between hr 18 and either hr 0 or hr 11 follicular fluid (Hydroxyphenylacetate, 3-Hydroxyisovalerate, Cysteine, Phosphorylethanolamine, Glutamate; Figure 3B).

**FIGURE 4:**
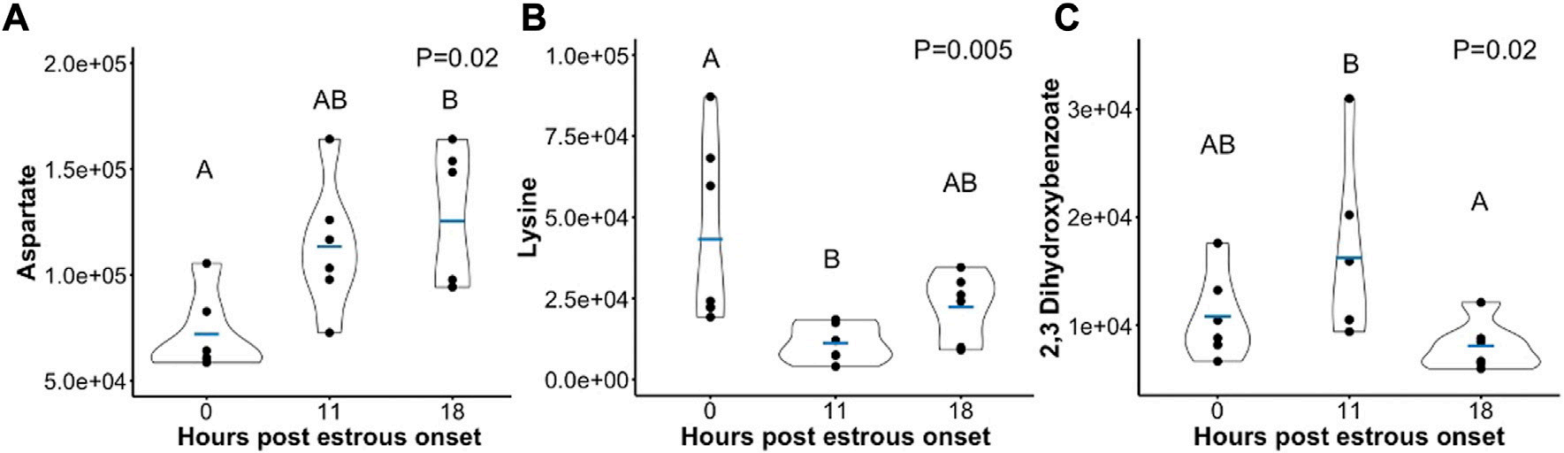
Scatterplots depicting peak area (*y*-axis) of metabolites that either **(A)** rose in abundance from hr 0 to hr 18 **(B)** dropped in abundance from hr 0 to hr 11, or **(C)** dropped in abundance from hr 11 to hr 18 post estrous onset. Mean metabolite abundance at timepoints with different letters are statistically different (Tukey’s Honest Significant Difference *post hoc* test *p* ≤ 0.05).

Few metabolites influenced by time post estrous onset followed a different trend in abundance. Aspartate was elevated at hr 18 compared to onset of estrus (Figure 4A). Though, a consistent rise in Aspartate was observed there were no statistical differences between hr 0 and 11 or hr 11 and 18 follicular fluid Aspartate level. Lysine decreased in abundance between hr 0 and 11 post first standing mount, but quantity of follicular fluid Lysine was no different between hr 18 and hr 0 or 11 (Figure 4B). Two, 3-Dihydroxybenzoate dropped in abundance from hr 11 to hr 18, but did not differ between hr 0 and either 11 or 18 (Figure 4C).

### 3.3 Pathway enrichment analysis of preovulatory follicular fluid metabolites influenced by time post estrous onset

Pathway analyses of all follicular fluid metabolites influenced by time post estrous onset revealed enrichment of 6 KEGG pathways, including “Alanine, Aspartate, and Glutamate Metabolism”, “Arginine Biosynthesis”, “D-Glutamine and D-Glutamate Metabolism”, “Citrate Cycle (TCA Cycle)”, “Aminoacyl-tRNA Synthesis”, and “Glyoxylate and Dicarboxylate Metabolism” (Table 2). Pathway analyses of only metabolites that rose in abundance from hr 0 to hr 11 or that rose in abundance from hr 0 to 11 and then decreased in abundance from hr 11 to 18 demonstrated similar enriched pathways including “Citrate Cycle (TCA Cycle)”, “D-Glutamine and D-Glutamate Metabolism”, “Alanine, Aspartate, and Glutamate Metabolism”, and “Glyoxylate and Dicarboxylate Metabolism” (Table 2). “Arginine Biosynthesis” and “Butanoate Metabolism” pathways were also enriched with metabolites that had increased abundance from hr 0 to 11 followed by decreased abundance from hr 11 to 18 (Table 2). Though multiple metabolic pathways were enriched with metabolites whose abundance varied across timepoint, relatively few metabolites contributed to observed enrichment. Pyruvate, Alpha-Ketoglutarate, Aspartate, Glutamate, Cysteine, Aconitate, and Lysine were the metabolites influenced by time post estrous onset that appeared in KEGG pathways identified by enrichment analysis.

**TABLE 2:** KEGG pathways significantly enriched with preovulatory follicular fluid metabolites that differed in abundance among collection times post estrous onset.

**Pathway**	**Pathway name**	^*^ **Match status**	**FDR**	**Differentially abundant metabolites in pathway**
bta00250	Alanine, Aspartate and Glutamate Metabolism	^a^4/28 ^bc^3/28	^a^0.020 ^b^0.076 ^c^0.040	^abc^C00026 (Alpha-Ketoglutarate) ^a^C00049 (Aspartate) ^abc^C00025 (Glutamate) ^abc^C00022 (Pyruvate)
bta00220	Arginine Biosynthesis	^a^3/14 ^c^2/14	^a^0.020 ^c^0.094	^ac^C00026 (Alpha-Ketoglutarate) ^a^C00049 (Aspartate) ^ac^C00025 (Glutamate)
bta00471	d-Glutamine and d-Glutamate Metabolism	^abc^2/5	^a^0.030 ^b^0.043 ^c^0.030	^abc^C00026 (Alpha-Ketoglutarate) ^abc^C00025 (Glutamate)
bta00020	Citrate Cycle (TCA Cycle)	^abc^3/20	^a^0.030 ^b^0.043 ^c^0.028	^abc^C00026 (Alpha-Ketoglutarate) ^abc^C00022 (Pyruvate) ^abc^C00417 (Aconitate)
bta00970	Aminoacyl-tRNA Biosynthesis	^a^4/48	^a^0.033	^a^C00049 (Aspartate) ^a^C00025 (Glutamate) ^a^C00097 (Cysteine) ^a^C00047 (Lysine)
bta00630	Glyoxylate and Dicarboxylate Metabolism	^abc^3/32	^a^0.080 ^b^0.084 ^c^0.044	^abc^C00417 (Aconitate) ^abc^C00025 (Glutamate) ^abc^C00022 (Pyruvate)
bta00650	Butanoate Metabolism	^c^2/15	^c^0.094	^c^C00026 (Alpha-Ketoglutarate) ^c^C00025 (Glutamate)

^*^ Number of differentially abundant metabolites in pathway/total number of metabolites in pathway.

^a^ data representative of pathway enrichment analysis using all metabolites that differed in abundance among timepoints.

^b^ data representative of pathway enrichment analysis using metabolites that increased in abundance from h 0 to h 11.

^c^ data representative of pathway enrichment analysis using metabolites that increased in abundance from h 0 to h 11 and also decreased in abundance from h 11 to h 18.

### 3.4 Preovulatory follicular fluid metabolites influenced by FF E2:P4 ratio classification

Eighteen preovulatory follicular fluid metabolites differed in abundance based on follicular fluid E2:P4 ratio classification (Table 3). Fourteen of these metabolites also differed in abundance among collection timepoints post estrous onset (myo-Inositol, Lactate, Pyruvate, N-acetyl-D-glucosamine 6-phosphate, Alpha-Ketoglutarate, Cholate, Glycerol-3-phosphate, Hydroxyphenylacetate, Aspartate, 2,3 Dihydroxybenzoate, Aconitate, Kynurenine, Orotate, and Phosphorylethanolamine; Table 1; Table 3). Similar to the results from analysis of metabolites based on time post estrous onset, a majority of metabolites that differed among FF E2:P4 ratios rose or tended to rise in abundance as FF E2:P4 ratio decreased from values expected near the onset of estrus to values expected nearing 11 h after first standing mount (myo-Inositol, Lactata, Pyruvate, N-acetyl-D-glucosamine 6-phosphate, Alpha-Ketoglutarate, Cholate, Glycerol-3-phosphate, Hydroxyphenylacetate, Aconitate, Kynurenine, Orotate, Phosphorylethanolamine). One distinction between analyses based on time post estrous onset and FF E2:P4 ratio was that myo-Inositol, Lactate, N-acetyl-D-glucosamine 6-phosphate, Cholate, and Kynurenine all dropped in abundance from hr 11 to hr 18 in the analysis based on time, whereas these metabolites did not significantly decrease in abundance between FF E2:P4 classifications indicative of approximately 11 and 18 h post estrous onset (Table 1; Table 3). As with the analysis based on time post estrous onset, Aspartate was elevated in the FF E2:P4 classification expected at hr 18 compared to onset of estrus (Table 3).

**TABLE 3:** Preovulatory follicular fluid metabolites that varied in abundance among follicular fluid E2:P4 ratio classifications.

**Metabolite**	**^†^** **Least squares means**			
	**0**	**11**	**18**	* **P**
Hydroxyphenylacetate	92,331 ± 4,806^a^	163,878 ± 3,816^b^	109,006 ± 4,109^a^	<0.001
Aspartate	77,830 ± 3,687^a^	101,532 ± 4,213^ab^	137,786 ± 3,687^b^	0.001
N-acetyl-D-glucosamine 6-phosphate	27 ± 149^a^	1,592 ± 171^b^	228 ± 199^ab^	0.003
Pyruvate	4,564,570 ± 171,529^a^	6,997,512 ± 192,971^b^	4,637,113 ± 192,971^a^	0.006
Alpha-Ketoglutarate	42,689 ± 3,548^a^	91,608 ± 3,992^b^	43,466 ± 3,992^a^	0.006
Glycerol-3-phosphate	2,517 ± 1,019^a^	12,952 ± 891^b^	7,598 ± 891^Ab^	0.007
myo-Inositol	159,336 ± 51,110^a^	647,828 ± 58,411^b^	567,821 ± 51,110^ab^	0.008
Lactate	30,895,067 ± 3,202,428^a^	66,276,567 ± 3,659,918^b^	56,820,210 ± 3,202,428^ab^	0.009
Phosphorylethanolamine	13,673 ± 3,310^a^	24,237 ± 3,782^Ab^	51,496 ± 3,310^b^	0.012
Orotate	964 ± 305^a^	5,779 ± 346^b^	836 ± 370^a^	0.012
Jasmonate	15,988,962 ± 19,434^a^	15,888,480 ± 31,094^ab^	15,734,453 ± 19,434^b^	0.014
2,3-Dihydroxybenzoate	11,125 ± 458^ab^	14,255 ± 523^a^	8,266 ± 523^b^	0.017
Succinate/Methylmalonate	1,488,465 ± 4,879^a^	1,551,601 ± 7,318^b^	1,486,125 ± 5,489^a^	0.021
Aconitate	39,475 ± 1,160^ab^	51,923 ± 1,590^A^	35,550 ± 1,488^b^	0.029
Cholate	27,381 ± 3,592^a^	62,050 ± 3,143^b^	39,536 ± 3,143^ab^	0.034
Cystathionine	9,825 ± 1,223^a^	18,744 ± 1,070^A^	8,203 ± 1,426^a^	0.034
Homocitrulline	223 ± 43^a^	644 ± 37^b^	545 ± 37^ab^	0.035
Kynurenine	10,805 ± 1,388^a^	21,616 ± 1,388^b^	11,805 ± 1,388^ab^	0.044

^†^ Least squares mean ± standard error of the mean for metabolite peak area.

^*^ P value reported for main effect of FF E2:P4 ratio classification.

^ab^ P ≤ 0.05.

^aA or bB^P ≤ 0.10. Pairwise comparison p values were adjusted using Tukey's Honest Significant Difference post hoc test.

### 3.5 Pathway enrichment analysis of preovulatory follicular fluid metabolites influenced by FF E2:P4 ratio

The KEGG pathway “Citrate Cycle (TCA Cycle)” was enriched in pathway analyses including all metabolites that differed among FF E2:P4 classifications, metabolites that rose from FF E2:P4 classification indicating onset of estrus to FF E2:P4 classification indicating hr 11, and metabolites that rose from FF E2:P4 classifications indicating hrs 0 and 11 but also decreased from FF E2:P4 classifications indicating hrs 11 and 18 (Table 4). The pathway “Alanine, Aspartate, and Glutamate Metabolism” was enriched with metabolites that differed among FF E2:PR ratio classifications and metabolites that rose from FF E2:P4 classifications indicating hrs 0 and 11 but also decreased from FF E2:P4 classifications indicating hrs 11 and 18 (Table 4). Three additional pathways including “Glyoxylate and Dicarboxylate Metabolism”, “Cysteine and Methionine Metabolism”, and “Glycine, Serine and Threonine Metabolism” were also enriched with metabolites that rose from FF E2:P4 classifications indicating hrs 0 and 11 and decreased from FF E2:P4 classifications indicating hrs 11 and 18 (Table 4). As observed in the primary analysis based on time post estrous onset, multiple metabolic pathways were enriched with metabolites whose abundance varied across FF E2:P4 ratio classifications and relatively few metabolites contributed to observed enrichment. Pyruvate, Alpha-Ketoglutarate, Aconitate, Aspartate, and Cystathionine were the metabolites influenced by FF E2:P4 ration classification that appeared in KEGG pathways identified by enrichment analysis.

**TABLE 4:** KEGG pathways significantly enriched with preovulatory follicular fluid metabolites that differed in abundance among follicular fluid E2:P4 ratio classifications.

**Pathway**	**Pathway name**	^*^ **Match status**	**FDR**	**Differentially abundant metabolites in pathway**
bta00020	Citrate Cycle (TCA Cycle)	^abc^3/20	^a^0.055 ^b^0.044 ^c^0.002	^abc^C00026 (Alpha-Ketoglutarate) ^abc^C00022 (Pyruvate) ^abc^C00417 (Aconitate)
bta00250	Alanine, Aspartate and Glutamate Metabolism	^a^3/28 ^c^2/28	^a^0.076 ^c^0.079	^ac^C00026 (Alpha-Ketoglutarate) ^a^C00049 (Aspartate) ^ac^C00022 (Pyruvate)
bta00270	Cysteine and Methionine Metabolism	^c^2/33	^c^0.079	^c^C00022 (Pyruvate) ^c^C02291 (Cystathionine)
bta00630	Glyoxylate and Dicarboxylate Metabolism	^c^2/32	^c^0.079	^c^C00417 (Aconitate) ^c^C00022 (Pyruvate)
bta00260	Glycine, Serine and Threonine Metabolism	^c^2/34	^c^0.079	^c^C00022 (Pyruvate) ^c^C02291 (Cystathionine)

^*^ Number of differentially abundant metabolites in pathway/total number of metabolites in pathway.

^a^ Data representative of pathway enrichment analysis using all metabolites that differed in abundance among FF E2:P4 classifications.

^b^ Data representative of pathway enrichment analysis using metabolites that increased in abundance from FF E2:P4 ratio classification expected at h 0 to FF E2:P4 ratio classification expected at h 11.

^c^ Data representative of pathway enrichment analysis using metabolites that increased in abundance from FF E2:P4 ratio classification expected at h 0 to FF E2:P4 ratio classification expected at h 11 and also decreased in abundance from FF E2:P4 ratio classification expected at h 11to FF E2:P4 ratio classification expected at h 18.

## 4 Discussion

**FIGURE 5:**
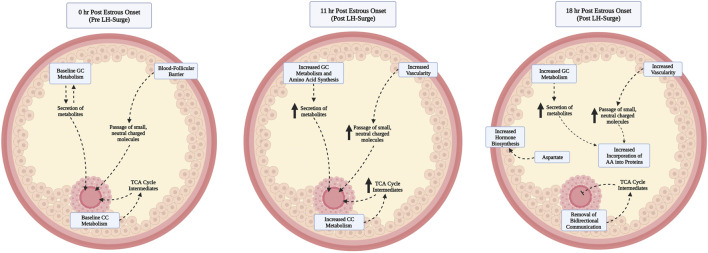
Proposed model of contributors to and functional relevance of elevated levels of follicular fluid metabolites at hr 11 vs. 0 post onset of estrus. As the preovulatory follicle launches an early response to the luteinizing hormone surge, granulosa and cumulus cells increase metabolism, amino acids are produced, and increased blood follicle barrier permeability leads to increased bioavailability of metabolites to be shuttled to the oocyte before breakdown of gap junctional transfer between the cumulus and oocyte, readily used by the cumulus for expansion, or to be used by hr 18 post estrous onset for incorporation into proteins. By hr 18, the follicular fluid metabolite milieu remains similar to that of hr 11, with the exception of decreased abundance of a small number of metabolites that are hypothesized to have been utilized by the follicular cells and oocyte during previous hours or whose production has slowed such that usage exceeds metabolite accumulation.

This study is the first of our knowledge to interrogate preovulatory follicular fluid metabolome profiles to determine metabolites whose abundance differed based on time post estrous onset in cattle. As somatic follicular cell differentiation and oocyte maturation commence following the LH surge, it is not surprising that preovulatory follicular fluid metabolome profiles at hr 0 (assumed pre-LH surge) and hr 11 (assumed post-LH surge) were distinct. Pyruvate, Alpha-Ketoglutarate, and Glutamate all varied in abundance according to time post estrous onset. These metabolites were the key contributors to significant pathway enrichment analysis in each KEGG pathway identified. The combination of metabolite enrichment in multiple metabolic related KEGG pathways and the change in abundance of key metabolites in glucose metabolism, the TCA cycle, and amino acid synthesis throughout post estrous follicle differentiation demonstrates essential shifts in intrafollicular metabolism during the hours leading up to and following the LH surge (Figure 5). In all of the above metabolites, greatest abundance was observed at 11 h after the first standing mount.

A rise in follicular fluid metabolites involved in energy and amino acid production during the early LH surge response is in line with increased follicular metabolism and need for such byproducts to support the energetically demanding transformations in the oocyte, cumulus, and granulosa cells in response to the LH surge (Figure 5). The transition of granulosa cells from proliferative function pre-LH surge to elevated metabolism, amino acid production, and increased vascularity at 6 h post surge (Gilbert et al., 2011) may partly explain the rise in many follicular fluid metabolites from hr 0 to hr 11 in the current study. Though differences in follicular progression from hr 6 post LH surge to hr 11 post estrous onset must be considered, follicular samples collected at hr 11 after first standing mount should be within approximately 7 h of the peak LH surge and thus quite similar in developmental progression as those collected at 6 h post LH surge. Since follicular fluid composition is heavily influenced by secretions of granulosa cells, increased metabolic activity and amino acid synthesis of granulosa cells likely contributes to the increased abundance of metabolites such as Pyruvate, Alpha-Ketoglutarate, Aspartate, Glutamate, Cysteine, Aconitate, and additional amino acids. Increased vascularity of the ovulatory follicle due to granulosa cell expression of vascular endothelial growth factor in response to LH surge (Schams et al., 2001) following estrous onset may have also positively influenced follicular fluid metabolite abundance via increased opportunity for transfer of small, neutral to slightly negatively charged molecules via the blood follicle barrier (Cran et al., 1976; Shalgi et al., 1977; Hess et al., 1998). All metabolites found to differ among time points post estrous onset were <1 kDA in mass, and all had a net charge or 0 or -1 except myo-Inositol which had a net charge of −6. Though much remains to be discovered about the selectivity and time dependent permeability of the blood follicle barrier, composition of follicular fluid is influenced by passing of systemic molecules through this barrier and both follicular invasion of blood vessels and blood follicle barrier permeability have been reported to increase following the LH surge (Duffy et al., 2018).

An increase in follicular fluid metabolites following the LH surge could certainly also hold a functional role in metabolic supportive of cumulus expansion and final metabolic preparation of the oocyte for maturation, fertilization, and early embryonic cell divisions. Undoubtedly, the metabolic contributions of the somatic follicular components support oocyte energy production prior to the LH surge and breakdown of cumulus-oocyte connections (Sutton-McDowall et al., 2004; Thompson et al., 2007; Sutton-McDowall et al., 2010). This support, however, is likely equally important in the early hours post LH surge. Since disconnection of the oocyte and cumulus was first reported at approximately 9–12 h after peak LH surge (Hyttel et al., 1986), samples collected at hr 11 in the current study should represent follicular fluid encasing intact cumulus oocyte complexes or those early in the process of cumulus expansion and gap junctional breakdown. The oocyte relies on oxidative phosphorylation using substrates derived from cumulus cell glycolysis to metabolize glucose (Thompson et al., 2007). Therefore, elevated abundance of follicular fluid Pyruvate, Alpha-Ketoglutarate, Aspartate, Glutamate, Lactate, Cysteine, and Aconitate at hr 11 vs. 0 may be partially produced by elevated cumulus metabolism and metabolic transfer to the oocyte and surrounding follicular fluid. Elevations of such metabolites from granulosa metabolism and systemic uptake may also serve a supportive role in oocyte metabolism by transfer through the cumulus or direct uptake by the oocyte.

Interestingly, metabolites involved in glucose metabolism and TCA cycle pathways have previously been identified by our lab to differ in abundance based on preovulatory follicle developmental status prior to a LH surge induced by exogenous GnRH administration (Read et al., 2021; Read et al., 2022). Follicles with higher estradiol production or larger diameter immediately prior to GnRH administration contained greater quantity of Pyruvate, Alpha-ketoglutarate, Glutamate, and other TCA cycle intermediates than did their less developed counterparts. Increased abundance of metabolic compounds in the follicular fluid of developmentally superior follicles is hypothesized to contribute to the superior fertility and oocyte developmental competence for embryo production in such animals. In fact pathways of “Alanine, aspartate, and glutamate metabolism”, “Arginine biosynthesis”, “D-Glutamine and D-Glutamate Metabolism”, “Citrate cycle (TCA cycle)”, and “Aminoacyl-tRNA synthesis” which were enriched with metabolites influenced by time post estrous in the current study were previously reported to be enriched with metabolites increased in physiologically superior follicles that enclosed an oocyte with greater abundance of ATP (Read et al., 2021; Read et al., 2022). The detection of metabolites enriched within glucose and amino acid metabolism related pathways in both the current study and past complementary studies emphasizes the intrafollicular metabolic milieu directly leading up to and following the LH surge as essential for fertility in cattle.

In addition to the increased bioavailability of glucose metabolism, TCA cycle, and amino acid metabolites from hr 0 to 11, myo-Inositol increased from hr 0 to hr 11 and then decreased from hr 11 to hr 18. Myo-Inositol is critical for numerous cellular functions through its role in cell signaling pathways, such as oocyte maturation and fertilization, that rely on intracellular calcium release (Berridge, 1993; Whitaker, 2006). Indeed, increased levels of follicular fluid myo-Inositol and *in vitro* supplementation of myo-Inositol in oocyte maturation media were related to or improved oocyte maturation and ability to produce a quality embryo (Pesty et al., 1994; Holm et al., 1999; Chiu et al., 2002). An increased level of myo-Inositol in hr 11 follicular fluid likely serves similar function to the elevated abundance of amino acids and energetic metabolites at this time point for oocyte final preparation to complete the essential process of meiotic maturation. Additionally, functions of myo-Inositol to combat oxidative stress and reduce abundance of free radicals such as hydrogen peroxide could contribute to maintenance of an oxidatively balanced follicular environment when follicular metabolism in maximized at hr 11 post estrous onset (Jiang et al., 2013; Mohammadi et al., 2019).

It is interesting that hr 18 preovulatory follicular fluid samples were not distinct from hr 0 or hr 11 when sPLSDA was performed. Regardless, a small number of metabolites, especially those that were amino acids or were involved in glucose metabolism or the TCA cycle, differed in abundance between hr 11 and 18 samples. Decrease in select metabolite levels from hr 11 to hr 18 suggests a potential slowing of the cellular production or blood follicle barrier uptake of those specific metabolites by the follicle or a ratio of follicular metabolite usage that strongly exceeds production. Previous studies have suggested that the increased bioavailability of glucose metabolism byproducts and amino acids in the follicular fluid as part of an early to intermediate response to the LH surge may hold multiple roles in the preovulatory follicle including shuttle to the oocyte while gap junctions remain intact (Eppig et al., 2005; D'Aniello et al., 2007) and subsequent usage in later hours post LH surge for protein synthesis (Gilbert et al., 2011). By the time follicles in the current study reached hr 18 post estrous onset, amino acids or metabolic byproducts could have been utilized to meet granulosa and/or oocyte needs for developmental progression. Such metabolites could be lower in abundance at hr 18 vs. hr 11 because they had been previously utilized and were no longer as necessary for follicular processes required for fertility. Though most metabolites did not differ in abundance between hr 0 and hr 18 samples, Aspartate increased from hr 0 to hr 18. Higher Aspartate levels in hr 18 follicular fluid supports the previous results of others indicating increased transcripts for granulosa cell preparation for ovulation and further luteal function at 22 h after the LH surge (Assidi et al., 2010). Aspartate has previously been implicated to function in endocrine tissues to promote hormone biosynthesis and may be functionally relevant to promote progesterone production in granulosa cells approaching ovulation (D'Aniello et al., 2000; D'Aniello et al., 2007). Further, potentially elevated Aspartate levels in ovulated follicular fluid may have positive impacts on the sperm quality (D’Aniello et al., 2005).

One weakness of the current study is the variation of LH surge timing relative to estrous onset. It is generally accepted that the pre-ovulatory gonadotropin surge occurs near the onset of estrus in bovine [within ∼0.5–5 h (Mondal et al., 2006; Rajamahendran et al., 1989; Dieleman et al., 1986; Stevenson et al.)]. Some studies have indicated occurrence of the surge before estrus (Saumande and Humblot, 2005) or the onset of surge to occur before the onset of estrus (Rajamahendran et al., 1989). Timing of the LH surge could have been more controlled if animal were not allowed adequate time to display estrus and GnRH was administered to induce the LH surge. Estrous expression was the preferred method of LH surge induction and timing due to a strong relationship between preovulatory follicle status at GnRH administration to induce ovulation and animal fertility, oocyte competence, and the follicular fluid metabolome. Since the majority of studies indicate the LH surge to occur within 0.5–5 h post estrous onset, mild variation in LH surge timing relative to estrus was preferred to reduce variability in preovulatory follicle physiological status. Nevertheless, some cows may be more or less inclined to mount or stand to be ridden based on weather conditions, age, or dominance. Low inclination to be mounted could lead to an animal being detected in estrus hours after a more sexually active animal. In such instances, the LH surge could have occurred before the animal was visually detected in estrus. To add confidence in the current study, we performed a secondary analysis that classified follicular fluid samples based on FF E2:P4 ratio. Analysis of metabolome profiles of the FF E2:P4 ratio categorized samples followed a remarkably similar trend to those analyzed based on collection timepoint. This similarity further points to the dynamic changes in intrafollicular metabolite milieu as preovulatory follicles respond to the LH surge and prepare for ovulation.

## 5 Conclusion

Outcomes of the current study highlight the myriad of intrafollicular changes that occur following onset of estrus in cattle. Dynamic changes in abundance of metabolites from estrus to hr 11 post estrous onset highlight the essentiality of follicular metabolism in early response to the LH surge and pose metabolites of key interest for future studies. Continued efforts to elucidate the biological function of each metabolite’s rise and/or fall in abundance throughout oocyte maturation and follicle progression to ovulation will provide invaluable opportunity to 1 day improve the intrafollicular environment for oocyte maturation, better support the developing corpus luteum, provide more physiological culture conditions for *in vitro* oocyte maturation, and thus improve fertility in cattle and other mammalian species.

## Data Availability

The original contributions presented in the study are publicly available. This data can be found here: [https://doi.org/doi:10.5061/dryad.1vhhmgqz0].
